# Probable neuroimmunological link between *Toxoplasma *and cytomegalovirus infections and personality changes in the human host

**DOI:** 10.1186/1471-2334-5-54

**Published:** 2005-07-06

**Authors:** Martina Novotná, Jitka Hanusova, Jiří Klose, Marek Preiss, Jan Havlicek, Kateřina Roubalová, Jaroslav Flegr

**Affiliations:** 1 Department of Parasitology, Charles University, Viničná 7, Prague 128 44, Czech Republic; 2 Central Medical Psychological Unit, Central Military Hospital, U vojenské nemocnice 1200, Prague 169 02, Czech Republic; 3 Prague Psychiatric Clinic, Ústavní 91, Prague, 180 00, Czech Republic; 4 Department of Anthropology, Faculty of Humanities, Charles University, Husnikova 2075, 158 00 Prague 13, Czech Republic; 5 National Reference Laboratory for Herpesviruses, National Institute of Public Health, Šrobárova 48, Prague 100 42, Czech Republic

## Abstract

**Background:**

Recently, a negative association between *Toxoplasma*-infection and novelty seeking was reported. The authors suggested that changes of personality trait were caused by manipulation activity of the parasite, aimed at increasing the probability of transmission of the parasite from an intermediate to a definitive host. They also suggested that low novelty seeking indicated an increased level of the neurotransmitter dopamine in the brain of infected subjects, a phenomenon already observed in experimentally infected rodents. However, the changes in personality can also be just a byproduct of any neurotropic infection. Moreover, the association between a personality trait and the toxoplasmosis can even be caused by an independent correlation of both the probability of *Toxoplasma*-infection and the personality trait with the third factor, namely with the size of living place of a subject. To test these two alternative hypotheses, we studied the influence of another neurotropic pathogen, the cytomegalovirus, on the personality of infected subjects, and reanalyzed the original data after the effect of the potential confounder, the size of living place, was controlled.

**Methods:**

In the case-control study, 533 conscripts were tested for toxoplasmosis and presence of anti-cytomegalovirus antibodies and their novelty seeking was examined with Cloninger's TCI questionnaire. Possible association between the two infections and TCI dimensions was analyzed.

**Results:**

The decrease of novelty seeking is associated also with cytomegalovirus infection. After the size of living place was controlled, the effect of toxoplasmosis on novelty seeking increased. Significant difference in novelty seeking was observed only in the largest city, Prague.

**Conclusion:**

*Toxoplasma *and cytomegalovirus probably induce a decrease of novelty seeking. As the cytomegalovirus spreads in population by direct contact (not by predation as with *Toxoplasma*), the observed changes are the byproduct of brain infections rather than the result of manipulation activity of a parasite. Four independent lines of indirect evidence, namely direct measurement of neurotransmitter concentration in mice, the nature of behavioral changes in rodents, the nature of personality changes in humans, and the observed association between schizophrenia and toxoplasmosis, suggest that the changes of dopamine concentration in brain could play a role in behavioral changes of infected hosts.

## Background

The protozoan parasite *Toxoplasma gondii *specifically influences the behavior of intermediate hosts. Infected mice have impaired motor performance [1], deficits in learning capacity and memory [2], higher activity levels both in novel and familiar environments [3-7], lower ability of discriminating between familiar and novel surroundings [3,4,8], and longer reaction times [9]. Infected rats have higher activity levels [10], lower neophobia [11], reduced learning capacity [2] and reduced specific predator avoidance [12]. Humans with latent toxoplasmosis have significantly deteriorated psychomotor performance (prolonged simple reaction times) in comparison with *Toxoplasma*-negative subjects [13] and are at higher risk of traffic accidents [14]. Subjects with latent toxoplasmosis are known to show specific changes in some personality dimensions as measured by the 16PF questionnaire [15-17] and Cloninger's TCI (Temperament and Character Inventory) [18]. Recently, Flegr *et al*. [18] used changes in the TCI dimension novelty seeking for monitoring possible shifts in dopaminergic activity in the brain of infected subjects. Increase in dopamine levels in infected subjects was expected based on direct measurement of neurotransmitter levels in animals [19] and the reported positive correlation between schizophrenia and toxoplasmosis [20-25] or risk factors [26-28]. Same authors expect that the background level of dopamine in the basal ganglia negatively correlates with the personality dimension novelty seeking as measured with Cloninger's TCI questionnaire [29-33]. Therefore, the authors of previous study interpreted lower novelty seeking in the subset of *Toxoplasma*-infected subjects as an indirect indicator of increased dopamine levels in brain tissue. An important caveat of the published study was failure to adjust for size of place of residence. The risk of *Toxoplasma *infection in the Czech Republic is known to correlate negatively with size of place of residence [34]. Since several personality factors could also correlate with this variable, it is difficult to tell to what extent the negative correlation between novelty seeking and latent toxoplasmosis may be biased by the correlation between novelty seeking and size of place of residence and between size of place of residence and the risk of *Toxoplasma *infection. In this light, it would be of relevance to test the effect of another neurotropic pathogen with a different life cycle and transmission route on personality in infected humans. Human cytomegalovirus seems to be a suitable model for the study among the Czech population.

Both the human cytomegalovirus (CMV) and protozoon *Toxoplasma gondii *are extremely common human pathogens. Depending on hygiene standards and habits, socioeconomic parameters and population demographic structure, the prevalence rates of CMV and *Toxoplasma *in different countries vary between 40–100 % and 20–70 %, respectively. CMV is transmitted by close contact between infected subjects, via blood or blood products, sexual intercourse, or parentally. On the other hand, *Toxoplasma *is transmitted either by consumption of raw or undercooked meat with tissue pseudocysts or by ingestion of food or water contaminated with cat feces containing oocysts of the parasite. Infection of pregnant woman with CMV or *Toxoplasma*, especially in the first trimester of pregnancy, frequently results in severe damage to the nervous system of the foetus or abortion [35,36]. Postnatally acquired infections in immunocompetent subjects are probably life-long but usually harmless and asymptomatic. However, the latent CMV or *Toxoplasma *infections can be activated in immunocompromised patients, e.g. those with AIDS, or immunosuppressed transplant recipients [37-39]. Here, the activation may lead to dissemination of virus or *Toxoplasma *to various tissues, including those of the nervous system [40], with possible fatal outcome due to viral or toxoplasmic encephalitis. In encephalitis patients with toxoplasmosis the lesions are mainly localized in the hemisphere gray matter, thalamus and basal ganglia [41] while in patients with CMV encephalitis they are found mainly in the periventricular areas, hemisphere gray matter, cerebellum and spinal cord [40].

The main aim of the present study was to test whether the negative association between infection and novelty seeking can also be found for another very common neurotrophic pathogen, human cytomegalovirus, which is not transmitted by predation but by direct host-host contact. Another aim of the study was to establish whether the psychological effect of latent toxoplasmosis was evident after adjustment for an important confounder, i.e. size of place of residence. For these purposes, 533 conscripts previously tested for latent toxoplasmosis and examined with Cloninger's TCI questionnaire were screened for anti-CMV antibodies. Possible association between the two infections and TCI dimensions was analyzed.

## Methods

### Subjects

All personality testing was performed at the Central Military Hospital in Prague. Personality data were collected during regular psychological examinations of conscripts (men), aspirants to two military specializations, namely to those of military guards (guards) and military castle guards (presidential guards). All of the subjects were asked to voluntarily participate in the research project and to sign the informed consent form, if enrolled. About 80 % of the subjects consented to the use of their psychological test results for the research project purposes and provided 5 ml of blood for serological testing. The experimental subjects were recruited in two drafts in July 2000 and January 2001. Only those aged 19–21 were enrolled. By this we not only obtained a more age-homogeneous experimental set of 533 men but also excluded potential outliers, i.e., those who had avoided regular compulsory military service for more than 3 years and also all four subjects that declared higher (university) education. Reliable information on the size of place of residence was available for 299 enrollees, divided into three categories: those living in villages and small cities (<10 thousand population), those living in large cities (10–100 thousand population) and those living in the Czech capital, Prague (1.2 million population). The results of all analyses were virtually the same if the study subjects were divided into four categories (<10 thousand, 10–50 thousand, 50–100 thousand, >100 thousand). Similar results were also obtained if the categorization was done on the basis of size of the place where the subject had spent his childhood instead of that of his current place of residence. The recruitment of subjects and the data handling was in accordance with current rules in Czech legislation.

### Personality tests

Personality testing was performed with a computerized form of the TCI (Temperament and Character Inventory) [42] as translated by Kožený and Tisanská [43], with translation of two questions being modified and Cloninger's validation scale being substituted with Eysenck's Lie scale EP/R. The final questionnaire [44] contained 238 TCI items, including 12 Lie scale items. The subjects received written instructions to press the YES or NO key for particular items, depending on whether the sentence on the computer screen characterized or did not characterize their usual and most probable behavior or feelings in the given situations. The TCI questionnaire recognizes four dimensions of temperament, namely Harm avoidance (HA), Novelty seeking (NS), Reward Dependence (RD) and Persistence (PE), and three dimensions of character, i.e., Self-directedness (SD), Cooperativeness (C) and Self-transcendence (ST).

Testing of intelligence was performed with the Otis questionnaire [45]. The Otis test is a standard verbal intelligence test used by the Czech Army. It consists of 32 questions focused on understanding of the given relationships, linguistic sensitivity, and vocabulary skills. It requires definitions of the concepts as expressed by the closest descriptive characteristics, selection of concepts according to common characteristics of objects, choice of antitheses, rejection of disparate concepts and explanation of proverbs.

### Immunological tests for toxoplasmosis and CMV

All serological tests were carried out in the National Reference Laboratory for toxoplasmosis and the National Reference Laboratory for herpes viruses of the National Institute of Public Health, Prague. Specific anti *Toxoplasma *IgG and IgM antibody concentrations were determined by ELISA (IgG: SEVAC, Prague, IgM: TestLine, Brno), optimized for early detection of acute toxoplasmosis [46], and complement fixation test (CFT) (SEVAC, Prague) which is more sensitive and therefore more suitable for the detection of old *T. gondii *infections [47]. CFT titres of antibodies to *Toxoplasma *in sera were measured at dilutions between 1 : 8 and 1 : 1024. The subjects with negative results of IgM ELISA (positivity index<0.9) and CFT titres higher than 1 : 8 were considered latent-toxoplasmosis positive. Specific antiCMV IgG antibodies were measured by quantitative ELISA (ETI-CYTOK-G plus, DiaSorin). Antibody concentration was expressed in arbitrary units (AU). Individuals with AU < 40 were considered seronegative for CMV.

### Statistical analysis

The Statistica ^® ^v.6.0 was used for all statistical testing, i.e., for Log-linear analyses, ANOVA, ANCOVA, Kendall nonparametric test, and linear regression. The results of testing of ANCOVA assumptions, namely of the testing of normality of data distribution, normality of residuals and homogeneity of variances, were nonsignificant for all studied models.

## Results

**Figure 1:**
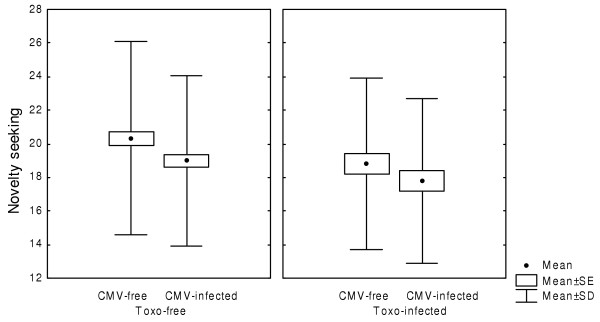
Novelty seeking in conscripts infected with cytomegalovirus and *Toxoplasma gondii.*

**Figure 2:**
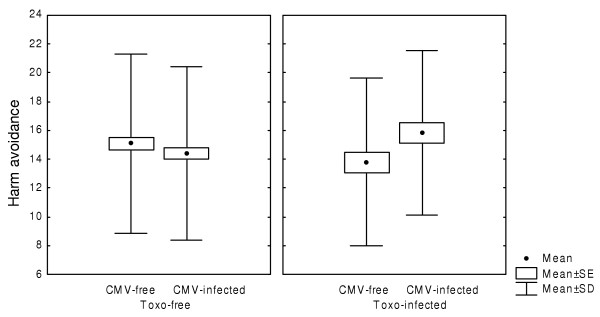
Harm avoidance in conscripts infected with cytomegalovirus and *Toxoplasma gondii.*

Five hundred thirty-three conscripts were tested for specific immunity against CMV and *Toxoplasma*. The prevalence rates of CMV and *Toxoplasma *infections were 50.5 % and 25.0 %, respectively. No statistical association was found between the two infections: CMV infection was detected in 48.9 % of *Toxoplasma*-infected and 51.0 % of *Toxoplasma*-free subjects. Log-linear analysis of the categorical factors *Toxoplasma *infection, CMV infection, education level and size of place of residence revealed just one significant interaction, namely the negative correlation between *Toxoplasma *infection and the size of place of residence. The difference between the models with and without this interaction was statistically significant (Chi^2 ^= 7.89, df = 2, P = 0.019). The results of two-way ANOVA with the factors *Toxoplasma *infection and CMV infection showed that both CMV infection (F_1,529 _= 7.37, P = 0.007) and *Toxoplasma *infection (F_1,529 _= 6.32, P = 0.012) were associated with lower novelty seeking scores (Fig. 1). *Toxoplasma *infection was also associated with higher self-transcendence scores (14.9 vs. 13.8, F_1,529 _= 4.52, P = 0.034). The results also showed a significant effect of the interaction between CMV and *Toxoplasma *infections on harm avoidance (F_1,529 _= 5.10, P = 0.024) (Fig. 2). Neither the effect of toxoplasmosis on self-transcendence nor the effect of the interaction between CMV and *Toxoplasma *infection on harm avoidance was significant after Bonferroni correction for twelve statistical tests (P = 0.408 and P = 0.284, respectively).

**Figure 3:**
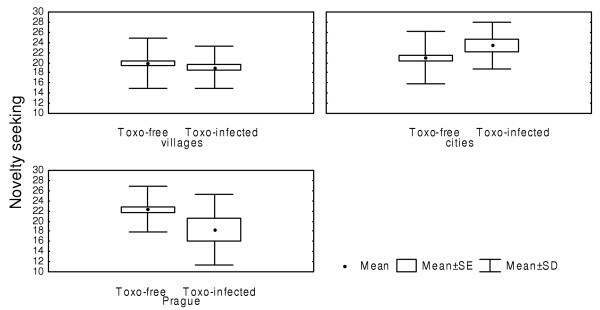
**Effects of toxoplasmosis and size of place of residence on the TCI dimension novelty seeking. **Villages of <10 000 population, cities of >10,000 and <100,000 population, Prague (1,200,000 population).

**Figure 4:**
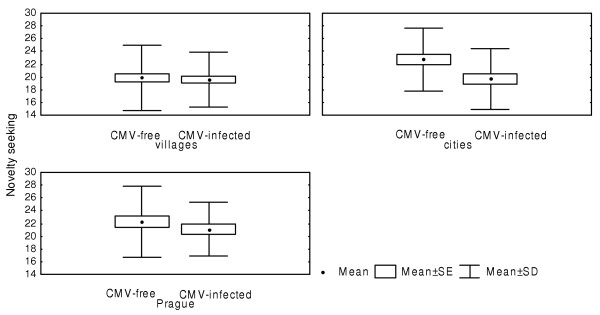
**Effects of CMV infection and size of place of residence on the TCI dimension novelty seeking. **Villages of <10 000 population, cities of >10,000 and <100,000 population, Prague (1,200,000 population).

Previous studies suggested that several confounders such as the level of education, size of place of residence, intelligence and motivation of experimental subjects could bias the TCI dimensions. Therefore, we repeated the analyses with the independent factors CMV infection, *Toxoplasma *infection, military profession (the factor probably correlated with the motivation of subjects), size of place of residence and intelligence as measured using the OTIS test (continuous variable). The results of this ANCOVA test concerning the association between novelty seeking and the CMV infections were similar to those obtained with the ANOVA test but the results of ANCOVA and ANOVA for *Toxoplasma *infection differed. CMV infected subjects were lower in novelty seeking (F_1,293 _= 7.80, P = 0.006). The differences in novelty seeking and self-transcendence between *Toxoplasma*-infected and *Toxoplasma*-free subjects fall below the formal level of statistical significance (F_1,293 _= 1.71, P = 0.19 for NS and F_1,293 _= 3.84, P = 0.051 for ST). At the same time, the analysis showed highly significant effect of interaction toxoplasmosis-size of place of residence on novelty seeking (F_2,293 _= 6.81, P = 0.001). *Toxoplasma *infection had only small impact on novelty seeking in the subjects living in settlements with less than 10 000 population (F_1,146 _= 0.77, P = 0.380), being associated with non-significantly higher novelty seeking scores in residents of the cities with 10–100 thousand population (F_1,79 _= 2.21, P = 0.142), and with low novelty seeking scores in Prague enrollees (F_1,66 _= 16.46, P = 0.0002) (Fig. 3). Similarly, association between CMV infection and novelty seeking was absent for inhabitants of villages and small cities (F_1,146 _= 0.59, P = 0.451), CMV infection being associated with lower novelty seeking scores for subjects living both in the cities with 10–100 thousand population and in Prague [(F_1,79 _= 7.75, P = 0.007), (F_1,66 _= 5.66, P = 0.021), respectively] (Fig. 4). The strength of effect of interaction between CMV and *Toxoplasma *infections on harm avoidance increased after adjustment for confounders (F_2,293 _= 7.98, P = 0.005; P = 0.06 after Bonferroni correction).

If the infection induces a shift in the personality dimension, the amount of personality shift should correlate with the concentration of specific antibodies. Such an effect (significant in one-tailed tests) was observed for both the CFT titre of anti-*Toxoplasma *(Kendal Tau = -0.14, P = 0.015) and the concentration of anti- CMV antibodies (F_1,229 _= 3.1, P = 0.079).

Lower intelligence scores and education level in *Toxoplasma*-infected subjects as reported previously were not observed for either CMV infection or toxoplasmosis in the present study. In fact, slightly lower intelligence was recorded in CMV-free than CMV-infected subjects (96.3 vs. 97.8, F_1,293 _= 1.452, P = 0.229). The significant effect of *Toxoplasma *infection on intelligence also disappeared after adjustment for the confounder size of place of residence (F_1,293 _= 0.003, P = 0.959).

## Discussion

Our results showed that CMV infected subjects had lower scores in the personality dimension novelty seeking than the CMV-free subjects. Novelty seeking in infected subjects negatively correlated with levels of anti-CMV antibodies. Negative correlation was also found between novelty seeking and *Toxoplasma*-infection in Prague, while in villages and smaller cities the correlation was not significant. The effect of both infections, although highly significant, was rather low, explaining about 2 % of total variability of the novelty seeking in our experimental set. The study also revealed several other significant effects CMV or *Toxoplasma *infections and their interaction on personality traits. While the effect of the infections on novelty seeking was *a priori *expected, other effects were revealed by an *ex post *analysis. Therefore, the results concerning the other effects have to be corrected for multiple statistical tests. After the Bonferroni's correction for multiple tests, no *ex post *revealed effect remained significant.

The lower values novelty seeking in the *Toxoplasma*-positive subjects suggest that infected men are on average more reflective, tend to require more detailed information when making an opinion and are not easily distracted. They are also more reserved, slow, controlled; they do not waste their energy and feelings. They tend to be organized, methodical, and prefer activities with strict rules and regulations. The association between latent toxoplasmosis and low novelty seeking scores has already been reported [18]. The present study showed that the relation between toxoplasmosis and novelty seeking was not caused by parallel correlations between the risk of *Toxoplasma *infection and size of place of residence and between size of place of residence and novelty seeking scores. Surprisingly, toxoplasmosis and CMV infections were associated with considerably lower novelty seeking scores only in large cities. There were no significant differences in novelty seeking between infected and pathogens-free subjects in settlements with fewer than 10 thousand population (CMV) or fewer than 100 thousand population (*Toxoplasma*). At present we have no explanation for this phenomenon.

A previous study performed on a larger set of experimental subjects revealed that *Toxoplasma*-infected subjects scored lower in verbal intelligence as measured with the Otis test and showed lower probability of achieving secondary education than *Toxoplasma*-free subjects [18]. It was demonstrated in several countries including the Czech Republic [34] that prevalence of toxoplasmosis is higher in villages and small cities than in larger cities. At the same time, the probability of achieving higher education and possibly also higher verbal intelligence could positively correlate with the size of place of residence. Therefore, the observed effects of toxoplasmosis can in fact reflect a parallel influence of size of place of residence (and corresponding life style) on a) the risk of acquiring *Toxoplasma *infection and b) probability of achieving secondary education and higher verbal intelligence. This model was strongly supported by the results of the present study. All associations between *Toxoplasma *infection and education level or intelligence disappeared after adjustment for the effect of place of residence. It is also noteworthy that CMV infection, which shows a similar prevalence rates in villages and small and large cities, has no association with education level or intelligence.

Neither the psychopathological antecedents nor the pre-infection personality data of the subjects were available for our analysis. Based on a case-control study, it is not possible to tell whether there is a causal relation between two statistically associated factors (e.g. infection and lower novelty seeking scores) and/or to determine the direction of such a relation. Theoretically, the infection could induce personality changes, personality factors may have influenced the risk of infection and possibly a third factor, such as the socioeconomic status, may have played a role in both personality dimensions and the risk of infection. In our view, however, the most parsimonious explanation is that the infection, more precisely the presence of pathogens in the brain of infected subjects, induces changes in neurotransmitter levels, causing in turn changes in TCI personality dimensions. This hypothesis is based on several lines of indirect evidence:

a) Two different neurotropic pathogens with quite different life cycles and transmission routes are associated with the same psychological effect. This makes the existence of a third factor responsible for both lower novelty seeking scores and higher risk of infection rather unlikely. Moreover, the existence of such a factor would cause a statistical association between *Toxoplasma *and CMV infections. However, CMV infection was detected with equal frequency among *Toxoplasma*-free and *Toxoplasma*-infected subjects.

b) The existence of correlation between novelty seeking scores and levels of specific antibodies (significant in one-tailed tests) was observed for both *Toxoplasma *and CMV. Such a correlation between the intensity of personality change and specific immunity could hardly be found if novelty seeking would influence the risk of infection or if an unknown factor would independently influence both novelty seeking and the risk of infection.

c) A decrease in neophilia in naturally neophilic mice and a decrease of neophobia of naturally neophobic rats, probably caused by lower ability of discriminating between familiar and novel surroundings, was also observed in animals experimentally infected with *Toxoplasma *[3,4,8], and a related behavioral effect (the apomorphine-mediated decrease of prepulse inhibition) was observed in rats infected with CMV [48]. Therefore, the direction of the causal relation between novelty seeking-related behavioral changes and *Toxoplasma*/CMV infections has already been experimentally established in rodents.

d) Lower novelty seeking scores are claimed by several authors [29-33] to correlate with the background level of dopamine in the basal ganglia. The increased level of dopamine was indeed observed in mice experimentally infected both with *Toxoplasma *[19] and CMV [49].

The specific neurological mechanism underlying the association between the infections and novelty seeking was not the subject of the present study. The available results of neuroimmunological studies, however, suggest that several cytokines engaged in inflammation processes, like interleukins 1, 2 and 6, directly influence the level and turnover of many neuromodulators, including dopamine [50-53]. An extremely low concentration of interleukin 2 (IL-2) is able to potentiate dopamine release evoked by number of different stimuli, including K^+ ^depolarization in mesencephalic cell cultures [54] and striatal slices [55]. Injections of IL-2 into the rat striatum have been shown to induce turning behaviors in rats that are typical of perturbation of the dopaminergic system [56] and subcutaneously administered IL-2 significantly increases locomotor activity of mice in the elevated plus-maze test [55]. Serious neuropsychiatric side effects demanding acute intervention regularly occur in oncological patients treated with IL-2 and lymphokine-activated killer cells [55,57,58]. Dormant forms of *Toxoplasma *and CMV persist in the brain of infected individuals for many years. The activation of acute disease in immunocompromised patients and immunohistochemical data obtained on mice [59] suggest that under normal conditions both diseases are kept in latent form by the host immune system. Local immune processes in the brain of subjects with latent infections are probably accompanied by local disturbances in particular cytokine levels [60-64]. This can influence the background level of neuromodulators and secondarily some of the personality dimensions.

It has been reported repeatedly that both toxoplasmosis and contact with cat, the definitive host of *Toxoplasma gondii*, are associated with higher risk of schizophrenia [20-28]. Similarly, schizophrenia was reported to correlate with CMV infection [65]. The background level of dopamine is supposed to play an important role in etiology of schizophrenia [66-68] and autoimmune-like effects on the dopamine system have been proposed as a possible mechanism involved in the pathogenesis of schizophrenia and Parkinson's disease [69]. Clinical investigations have detected increased levels of IL-2 in the CSF of schizophrenic patients manifesting symptoms of psychosis [70,71]. It can be speculated that the local inflammation-induced increase in dopamine in the brain of infected subjects can be in fact the missing link between schizophrenia and *Toxoplasma *or CMV infection.

## Conclusion

*Toxoplasma *and cytomegalovirus probably induce a decrease of novelty seeking of infected subjects. As the cytomegalovirus spreads in population by direct contact (not by predation as with *Toxoplasma*), the observed changes are the byproduct of brain infections rather than the result of manipulation activity of a parasite. Four independent lines of indirect evidence, namely direct measurement of neurotransmitter concentration in mice, the nature of behavioral changes in rodents, the nature of personality changes in humans, and the observed association between schizophrenia and toxoplasmosis, suggest that the changes of dopamine concentration in brain tissue could play a role in behavioral changes of infected hosts.

## Competing interests

The author(s) declare that they have no competing interests.

## Authors' contributions

MN and JF designated the study, wrote the manuscript and participated in analyses. JH, JK, JH and MP collected the data, participated in analyses and in manuscript writing. KR did serological analyses. All authors read and approved the final manuscript.

## Pre-publication history

The pre-publication history for this paper can be accessed here:


